# Tunable Fly Ash-Based Geopolymer Fibers for Multivariate Heavy-Metal Adsorption: Optimization and Mechanistic Insights

**DOI:** 10.3390/ma18204698

**Published:** 2025-10-13

**Authors:** Gongming Luo, Yuanbing Zhou, Shuangquan Liao, Sujitra Onutai

**Affiliations:** 1 A Key Laboratory of Advanced Materials of Tropical Island Resources of Ministry of Education, School of Materials Science and Engineering, Hainan University, Haikou 570228, China; 13929714549@163.com (G.L.); zhouyb@hainanu.edu.cn (Y.Z.); 2 Department of Materials Science, Faculty of Science, Chulalongkorn University, Bangkok 10330, Thailand; 3 Center of Excellence on Petrochemical and Materials Technology, Chulalongkorn University, Bangkok 10330, Thailand

**Keywords:** porous materials, geopolymer, composite fiber, adsorption, wastewater treatment, heavy-metal removal

## Abstract

This study presents the fabrication and performance optimization of porous fly ash-based geopolymer (FAGP)–polyethersulfone (PES) composite fibers with tunable FAGP loading for the multivariate adsorption of heavy-metal ions from aqueous solutions. Fibers containing 20 wt%, 40 wt%, and 60 wt% FAGP were prepared using phase inversion method and were characterized using X-ray computed tomography and mechanical testing. Adsorption experiments were conducted to assess the removal efficiencies of Pb^2+^, Cd^2+^, Cu^2+^, and Ni^2+^ at different pH values, temperatures, contact times, adsorbent dosage and initial metal-ion concentrations. The composite containing 60 wt% FAGP exhibited the high performance for all ions, and its performance was especially high for Pb^2+^. The isotherm and kinetic modeling revealed that the adsorption process followed Freundlich and Redlich–Peterson models, with mixed chemisorption–physisorption mechanisms depending on the metal-ion type. Compared with conventional adsorbents, the optimized composite fibers exhibited high adsorption capacity, enhanced handling suitability, and scalability in addition to their sustainability owing to the use of industrial by-products as precursors. These findings provide new insights into the structure–function relationships of FAGP composite fiber adsorbents and their potential for wastewater treatment applications.

## 1. Introduction

The contamination of global water resources is an escalating problem because water is essential for the survival of plants, animals, and humans. Pollutant discharges from the domestic and industrial sectors considerably contribute to the degradation of water quality [1]. Industrial wastewater contains various types of ions and molecules such as metal ions, surfactants, and dyes [2]. The heavy metals in wastewaters discharged from the rapidly developing metal plating, mining, fertilizer, and battery industries are directly or indirectly released into the environment [3,4]. Heavy metals are inorganic nonbiodegradable species. The primary heavy-metal ions found in wastewater are cadmium, copper, lead, and nickel [3]. All of them can affect the human and animal bodies. For example, lead destroys the metabolic system [5], copper causes acute and chronic disorders in humans [6,7], cadmium can cause abnormal glycosuria and proteinuria [8], and nickel causes lung and bone cancers [9]. Thus, wastewater should be treated before its release into the environment.

Sustainable wastewater treatment to mitigate global pollution has been extensively studied [10]. Common methods for heavy metals removal from wastewater include chemical precipitation, membrane filtration, ion exchange, biosorption, and adsorption [3,11,12]. Among them, adsorption is one of the most widely employed techniques for heavy-metal removal. The advantages of adsorption include simple operation and cost-effectiveness [13]. Currently, natural minerals, agricultural waste, or industrial by-products, such as ash, silica, mud, and soil, are used as adsorbent materials [14]. Geopolymers are environmentally friendly materials with a three-dimensional aluminosilicate network structure [15,16,17,18]. Numerous studies revealed that these materials immobilize heavy-metal ions [19,20,21]. Geopolymer materials can immobilize heavy-metal ions and serve as adsorbents for the removal of heavy-metal ions [15,22,23]. Geopolymer-based adsorbents have been synthesized using different raw materials via different methods [24,25].

In recent years, porous metakaolin-based geopolymer beads have been produced via three dripping techniques, which involve the use of polyethylene glycol and liquid nitrogen as well as ionotropic gelation [26]. The results showed that the three methods produced beads with a diameter of 2–3 mm. In addition, direct foaming was employed to produce four near-net-shaped foams using a benchmark metakaolin-based geopolymer formulation. To modify the porosity from meso- to ultra-macro-porosity, hydrogen peroxide and metallic silicon were used at different concentrations as blowing agents [27]. A suspension dispersion solidification process was used to synthesize slag-based porous inorganic polymer microspheres with a diameter of approximately 100 μm to be used for Pb^2+^ adsorption [28]. However, the direct use of geopolymers as adsorbents in wastewater treatment still faces some challenges such as their limited regeneration and scalability for industrial production.

To address these limitations, fiber-based adsorbents have gained increasing attention owing to their superior mechanical properties, ease of separation, and modular deployment potential. Unlike powder or bead-type adsorbents, composite fibers can be directly packed into columns or fabricated into modules, enabling scalable and reusable treatment systems with reduced risk of secondary particle release. This structural advantage provides a practical pathway from laboratory synthesis to industrial application. In a previous study, the feasibility of synthesizing porous fly ash-based geopolymer (FAGP) composite fibers using the phase inversion method was demonstrated, and the composite fibers achieved effective removal of several heavy-metal ions at a fixed fiber composition [29]. However, the structure–performance relationships, effect of the geopolymer content on the adsorption process, and underlying adsorption mechanisms are still not comprehensively explored.

The present work examined the multivariate adsorption performance under various environmental conditions and methodically optimized the FAGP loading (20 wt%, 40 wt%, and 60 wt%) in the PES matrix. Moreover, the fiber morphology and integrity were investigated based on advanced characterization, using X-ray computed tomography and tensile testing. The effects of the pH, temperature, initial metal-ion concentration, and adsorbent dosage on the adsorption performance were also evaluated based on several adsorption experiments. In addition, kinetic models and adsorption isotherms were used to clarify the governing mechanisms of each metal-ion. This work provides novel insights into the development of sustainable and scalable geopolymer-based fiber adsorbents, through a combined approach of fiber design enhancement, operational parameter analysis, and mechanistic modeling, with future studies planned to evaluate performance in real wastewater systems.

## 2. Experimental Procedure

### 2.1. Materials and Geopolymer Preparation

Fly ash was obtained from the Mae Moh power plant in Lampang Province, Thailand and used as the primary aluminosilicate precursor. An industrial-grade sodium silicate solution (SiO_2_/Na_2_O, molar ratio = 3) and a sodium hydroxide solution (10 M) were used as alkali activators. PES and N-methyl-2-pyrrolidone (NMP) served as the polymer matrix and solvent, respectively. Analytical-grade metal salts (Pb(NO_3_)_2_, Cd(NO_3_)_2_·4H_2_O, Cu(NO_3_)_2_·3H_2_O, and Ni(NO_3_)_2_·6H_2_O) were purchased from Nacalai Tesque Inc., Kyoto, Japan and used to prepare stock solutions of Pb^2+^, Cd^2+^, Cu^2+^, and Ni^2+^, respectively.

To synthesize the geopolymer precursor, sodium silicate and sodium hydroxide solutions were mixed at a 2.5:1 mass ratio and combined with fly ash to form a homogeneous paste (solid content = 65 wt%). The mixture was cast into molds, sealed, and cured at 60 °C for 24 h, followed by aging at 25 °C for 6 days. The hardened geopolymer was then washed with distilled water until a neutral pH (7 ± 0.5) was achieved. It was then dried at 60 °C and ground into fine powder for use in fiber fabrication.

### 2.2. Fabrication of the Geopolymer–PES Composite Fibers

The phase inversion method was used to fabricate the composite fibers. PES was dissolved in NMP to form a 30 wt% polymer solution. FAGP powder was then incorporated at different loading levels (20 wt%, 40 wt%, and 60 wt%) and continuously stirred for 24 h to ensure uniform dispersion. The resulting slurry was extruded through a cylindrical needle (diameter = 0.6 mm) into a coagulation bath containing deionized water at 25 °C. The phase separation process continued for 24 h to remove the residual solvent. Next, the solidified fibers were collected and dried at 60 °C for another 24 h.

### 2.3. Characterization of Composite Fibers

The chemical compositions of the FAGP powder and composite fibers were determined using X-ray fluorescence (XRF) spectroscopy (ZSX Primus II; Rigaku Corp., Tokyo, Japan). Samples were prepared by pressing the FAGP powder and composite fibers into aluminum rings to form pellets for analysis. X-ray computed tomography (X-ray CT) was employed as a three-dimensional imaging technique to investigate the internal morphology of the materials. The geopolymer fiber pellets were cut into 3 cm segments, and their internal structure was examined using high-resolution microcomputed tomography. Scanning was performed at 0.1° rotation steps to achieve high-resolution imaging of the fiber morphology.

The Archimedes method was used to determine the apparent specific volume of the composite fibers. The dried fibers were immersed in methanol until the mass stabilized, indicating equilibrium. The apparent specific volume (*V_a_*) was calculated using Equation (1) as follows:

(1) $$V_{a}=\frac{Ws-Ww}{Ws}\times \frac{1}{\left(\rho_{0}-d\right)}+\frac{1}{d},$$

where *Ws* is the dry sample mass (g), *Ww* is the sample mass in methanol (g), $\rho_{0}$ is the density of methanol (g/cm^3^), and *d* is the air density (g/cm^3^).

The tensile strength of the composite fibers was measured using a load measurement device (LTS-500N-S20; Minebea Co. Ltd., Tokyo, Japan) to assess their mechanical resistance. The gauge length for testing was set to 30 mm at a crosshead speed of 1.5 mm/s. Five specimens (length = 50 mm) of each sample were tested.

### 2.4. Adsorption Experiments

Stock solutions of Pb^2+^, Cd^2+^, Cu^2+^, and Ni^2+^ (concentration = 1000 mg/L) were prepared by dissolving appropriate amounts of Pb(NO_3_)_2_, Cu(NO_3_)_2_·3H_2_O, Cd(NO_3_)_2_·4H_2_O, and Ni(NO_3_)_2_·6H_2_O in distilled water. Mono-heavy-metal solutions with concentrations of 10, 20, 40, 60, 80, 100, and 120 mg/L were then prepared by diluting the stock solutions. The pH of each solution was adjusted using nitric acid (HNO_3_, 0.1 M) or sodium hydroxide (NaOH, 0.1 M) as required for each experimental batch. All adsorption experiments were performed in triplicate (n = 3) under identical conditions to ensure reproducibility.

To evaluate the effect of the geopolymer dosage on the adsorption performance, different masses of FAGP composite fibers (0.05–0.5 g) were added to mono-heavy-metal solutions (20 mg/L, 40 mL) containing Pb^2+^, Cd^2+^, Cu^2+^, and Ni^2+^. The experiments were conducted at a constant temperature of 25 °C, a solution pH of 5, and a shaking time of 24 h. The uptake percentage and adsorption capacity were calculated for each fiber dosage.

The effect of pH on the adsorption performance was also investigated. The mono-heavy-metal solutions (20 mg/L, 40 mL) containing Pb^2+^, Cd^2+^, Cu^2+^, and Ni^2+^ were tested at different pH values (1–5), using the FAGP composite fibers (0.1 g) at 25 °C and a shaking time of 24 h. The uptake percentage and adsorption capacity were determined at each pH condition.

To assess the temperature effect on the adsorption performance, the mono-heavy-metal solutions (20 mg/L, 40 mL) containing Pb^2+^, Cd^2+^, Cu^2+^, and Ni^2+^ were shaken at 25 °C, 35 °C, and 45 °C for 24 h using the composite fiber (0.1 g), and the uptake percentage and adsorption capacity were determined at each temperature.

Finally, the effect of the initial metal-ion concentration (10–120 mg/L) on the adsorption performance was determined at fiber dosage = 0.1 g, pH = 5, temperature = 25 °C, and shaking time = 24 h. The uptake percentage and adsorption capacity were measured at each metal-ion concentration.

After testing, the solutions were characterized using atomic absorption spectroscopy (AA-6300: Shimazu Corp., Kyoto, Japan). The removal efficiency (*E*) was calculated using Equation (2).

(2) $$Removal\;efficiency(E,\%)=\frac{C_{i}-C_{eq}}{C_{i}}\times 100\%,$$

where *C_i_* is the metal-ion concentration of the heavy-metal solution (mg/L) and *C_eq_* is the remaining equilibrium concentration (mg/L).

The amount of metal ions adsorbed at equilibrium (q_e_) was calculated using Equation (3).

(3) $$q_{e}(mg/g)=\frac{{(C}_{i}-C_{eq})V}{C_{i}},$$

where *C_i_* is the metal-ion concentration of the heavy-metal solution (mg/L), *C_eq_* is the concentration at equilibrium (mg/L), and *V* is the volume of the solution (L).

## 3. Results and Discussion

### 3.1. Fiber Properties and Morphology

The chemical compositions of FAGP powder and the composite fibers with different FAGP loadings were determined using X-ray fluorescence (XRF) spectroscopy, and the results are summarized in Table 1. The FAGP powder primarily consisted of SiO_2_ (44.80%), Al_2_O_3_ (16.30%), Fe_2_O_3_ (12.40%), and CaO (7.60%). The incorporation of FAGP powder into the PES matrix increased the concentrations of SiO_2_, Al_2_O_3_, Fe_2_O_3_, CaO, MgO, and Na_2_O in the composite fibers.

**Figure 1:**
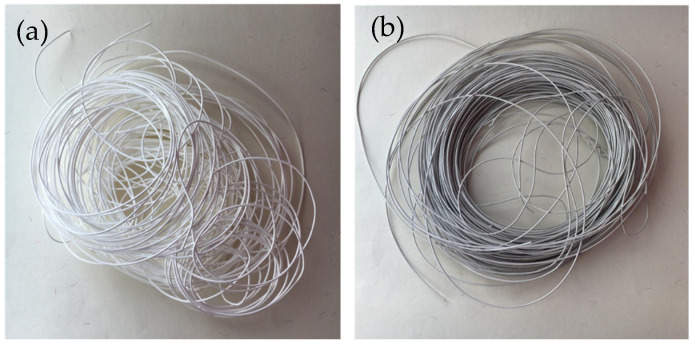
The sample of (**a**) PES fiber and (**b**) 60 wt% FAGP composite fiber.

Table 2 summarizes the properties of the composite fibers, PES fibers, and FAGP powder. An increase in the loading of FAGP powder increased the specific surface area of the composite fibers. The highest surface area (71.61 m^2^/g) was achieved with the incorporation of 60 wt% FAGP. In comparison, the surface areas of pure PES fibers, 20 wt% FAGP composite fibers, and 40 wt% FAGP composite fibers were 27.39, 50.05, and 57.50 m^2^/g, respectively. Moreover, mechanical testing confirmed that tensile strength decreased with increasing FAGP content, from 5.83 MPa for pure PES fibers (Figure 1a) to 1.40 MPa at 60 wt% FAGP loading (Figure 1b). Despite this reduction, a tensile strength of 1.40 MPa remains adequate for manual handling, column packing, and agitation during batch adsorption experiments.

**Figure 2:**
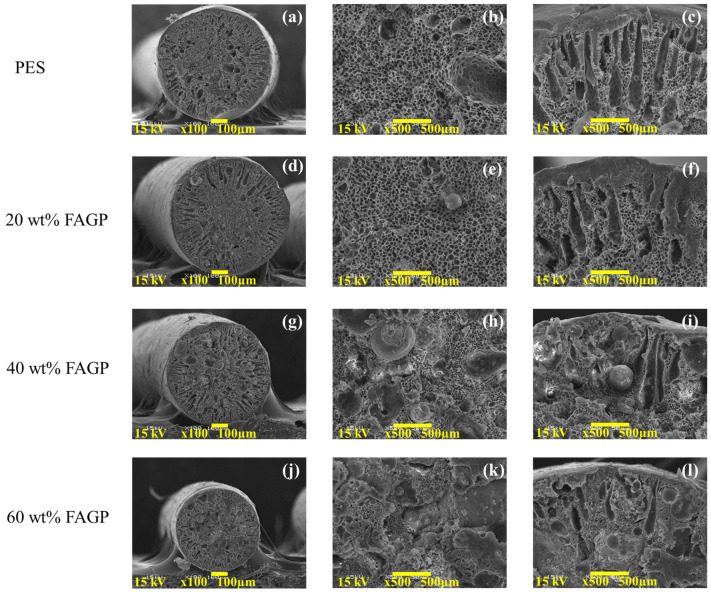
SEM images of the FAGP composite fibers with different amount of fly ash-based geopolymer. (**a**,**d**,**g**,**j**) show the cross-sectional morphology of fibers; (**b**,**e**,**h**,**k**) present the porous structure of the fibers; and (**c**,**f**,**i**,**l**) display finger-like pore of the fibers.

The morphological structures of the PES fiber and FAGP composite fibers are presented in Figure 2. The PES fibers (Figure 2a) and 20 wt% FAGP composite fibers (Figure 2d) exhibited diameters of approximately 500 µm. A slight reduction in fiber diameter was observed when the FAGP content exceeded 40 wt%, with the cross-sectional diameters of 40 wt% and 60 wt% FAGP composite fibers (Figure 2g,j) ranging from approximately 400 to 450 µm. The FAGP composite fibers exhibited a porous structure, with fly ash-based geopolymer powder dispersed within a polyethersulfone (PES) matrix. The outer region of the fibers displayed a finger-like pore structure, which gradually disappeared as the FAGP loading increased. The central region of the fibers showed a sponge-like porous structure. SEM images confirmed the distribution of geopolymer powder within the sponge-like core. Furthermore, the fibers became denser with higher FAGP loadings.

**Figure 3:**
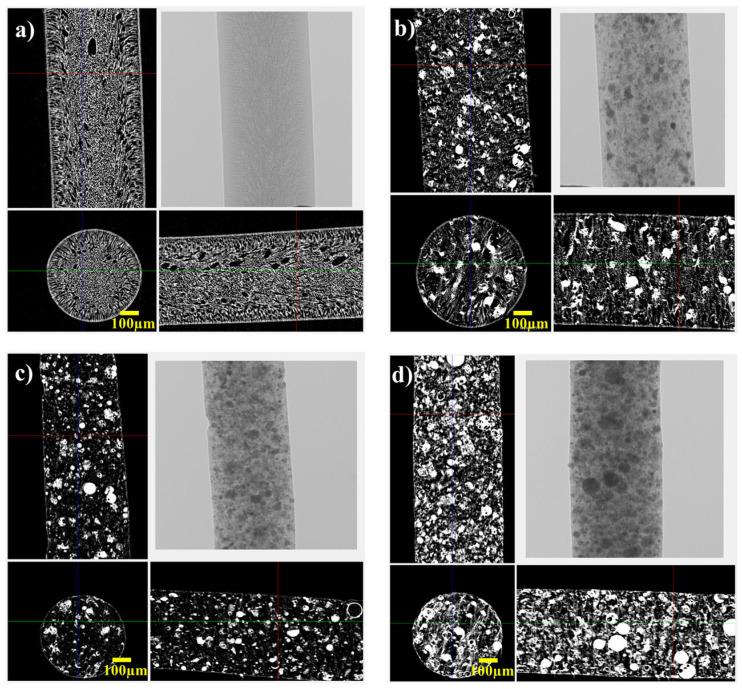
X-ray CT images of the FAGP composite fibers with different amounts of FAGPs: (**a**) PES, (**b**) 20% FAGP, (**c**) 40% FAGP, and (**d**) 60% FAGP.

X-ray CT was employed to assess the internal structure of the fibers (Figure 3). The X-ray CT images revealed the changes in the pore distribution with increasing the FAGP content. The 20 wt% FAGP fibers exhibited a uniform porous network, whereas the 60 wt% FAGP fibers exhibited increased density and occasional agglomeration of the FAGP powder. This densification is due to the limited dispersion capacity of the FAGP powder in the PES matrix at high FAGP loadings.

**Figure 4:**
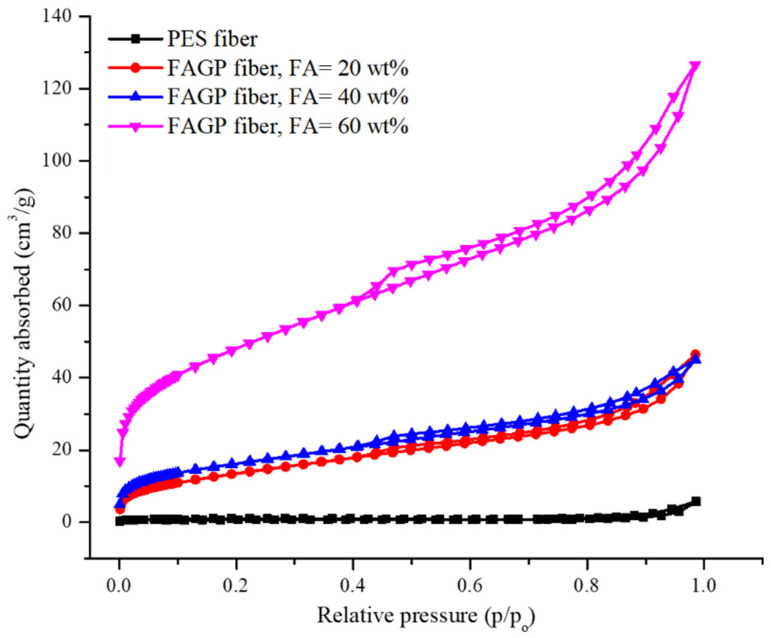
N_2_ isotherms of PES and the FAGP composite fibers.

Figure 4 shows the nitrogen adsorption–desorption isotherms of the PES and composite fibers at various relative pressures. The nitrogen adsorption capacity increased at higher loadings of the FAGP powder. A substantial increase in nitrogen adsorption was observed at relative pressures lower than 0.1. According to the International Union for Pure and Applied Chemistry classification, the shape of the isotherms, which is characterized by a hysteresis loop, corresponds to a type IV isotherm. These results confirm the mesoporous structure of the composite fibers.

### 3.2. Adsorption Performance Under Different Conditions

**Figure 5:**
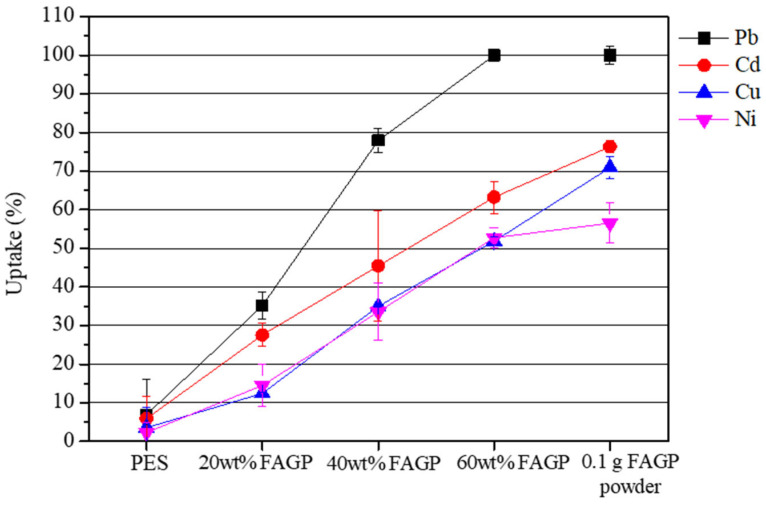
Preliminary adsorption screening of PES fiber, FAGP powder (0.1 g), and FAGP composite fibers with different FAGP loadings (20–60 wt%) for Pb^2+^, Cd^2+^, Cu^2+^, and Ni^2+^ (initial concentration = 20 mg/L, pH = 5, contact time = 24 h, FAGP composite fiber = 0.5 g and temperature at 25 °C).

Preliminary adsorption screening was carried out on PES fiber, FAGP powder (0.1 g), and composite fibers containing 20–60 wt% FAGP under identical conditions (initial concentration = 20 mg/L, pH = 5, contact time = 24 h, fiber dosage = 0.5 g, temperature = 25 °C). As shown in Figure 5, PES fibers displayed negligible adsorption, confirming that PES alone has little affinity for heavy metal ions. FAGP powder exhibited higher adsorption capacity, but its lack of mechanical integrity makes it unsuitable for use in filtration systems. Incorporating FAGP into PES fibers provided a balance between adsorption efficiency and structural stability. Adsorption performance improved progressively with increasing FAGP content, with the 60 wt% composite fibers showing the highest capacities for all tested ions, particularly Cd^2+^ and Pb^2+^. These findings indicate that the geopolymer fraction primarily governs adsorption efficiency, while the PES matrix contributes to strength and processability, thereby justifying the selection of 60 wt% FAGP fibers for detailed adsorption studies.

**Figure 6:**
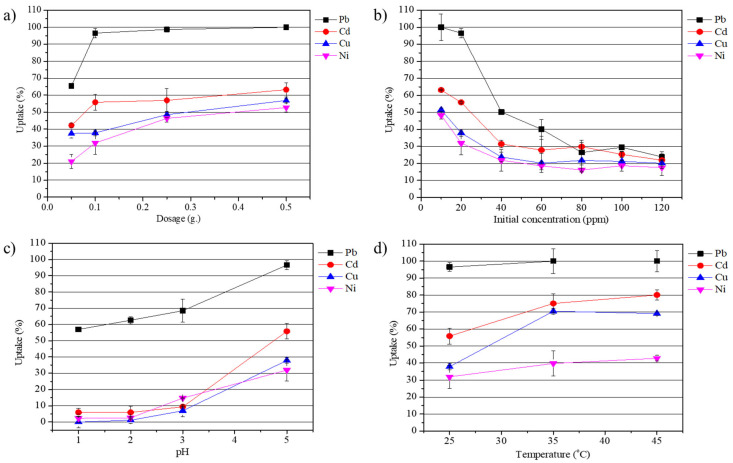
Parameters affecting heavy-metal adsorption on FAGP composite fibers: (**a**) fiber dosage, (**b**) initial concentration, (**c**) pH, and (**d**) temperature.

The effects of fiber dosage, initial metal-ion concentration, solution pH, and temperature on the adsorption behavior of heavy-metal ions on the composite fibers were systematically evaluated (Figure 6). The effect of the composite geopolymer fiber dosage (0.05–0.5 g) on the removal efficiencies of Pb^2+^, Cu^2+^, Cd^2+^, and Ni^2+^ is illustrated in Figure 6a. The removal efficiencies of Pb^2+^, Cu^2+^, Cd^2+^, and Ni^2+^ increased progressively with fiber dosage, reaching 100%, 56.88%, 63.22%, and 52.75%, respectively, at 0.5 g per 40 mL of solution. This indicates that the optimal dosage for Pb^2+^ removal is 0.5 g, at which complete removal was achieved. For Cu^2+^, Cd^2+^, and Ni^2+^, the same dosage provided only partial removal, suggesting that higher dosages would be required to reach complete adsorption under the tested conditions. Thus, while Pb^2+^ exhibits an optimal dosage of 0.5 g, the optimal dosages for Cu^2+^, Cd^2+^, and Ni^2+^ are higher than 0.5 g.

Figure 6b illustrates the effect of the initial metal-ion concentration (10–120 mg/L) on the removal efficiencies of Pb^2+^, Cu^2+^, Cd^2+^, and Ni^2+^. The results show that the removal efficiency of all ions gradually decreased with the increase in the initial metal-ion concentration. At an initial metal-ion concentration of 10 mg/L, approximately 100%, 51.45%, 63.13%, and 48.25% of Pb^2+^, Cu^2+^, Cd^2+^, and Ni^2+^, respectively, were removed. However, with the increase in the initial metal-ion concentration to 120 mg/L, the removal of all ions decreased. These findings indicate that at low initial metal-ion concentrations, the high availability of active adsorption sites on the fiber surface leads to enhanced removal efficiency. In contrast, at higher initial metal-ion concentration, the ratio of metal ions to available adsorption sites increases [23], resulting in incomplete adsorption, with high number of metal ions remaining in the solution. Industrial wastewater streams can contain heavy metals across a wide concentration range, from a few mg/L (e.g., plating rinse water [30,31,32,33]) to several hundred mg/L (e.g., mining effluents [34]). The concentration range investigated in this study (10–120 mg/L) was selected to capture mechanistic behavior under moderate loading conditions while ensuring measurable adsorption. Although this range does not cover all possible effluent scenarios, the results provide fundamental insights into adsorption kinetics and capacity. Future work will extend to real wastewater matrices containing multi-ion systems to further evaluate competitiveness under complex conditions.

The effect of solution pH on the removal efficiencies of Pb^2+^, Cu^2+^, Cd^2+^, and Ni^2+^ using the FAGP composite fibers is shown in Figure 6c. The lowest removal efficiency of all cations was observed at pH = 1, while gradual improvement was seen as the initial pH increased to 5. Specifically, the adsorption efficiencies of Pb^2+^, Cu^2+^, Cd^2+^, and Ni^2+^ increased from 56.94% to 96.57%, 0% to 37.83%, 5.90% to 55.81%, and 2.28% to 31.88%, respectively. At low initial pH values, the high concentration of H^+^ competes with metal cations for adsorption sites on the fibers, thereby reducing metal uptake. After adsorption, the equilibrium pH was generally lower than the initial value, which can be attributed to proton release during ion exchange and surface complexation reactions. As the initial pH increased, the extent of H^+^ competition decreased and the fiber surface likely became more negatively charged which may have enhanced electrostatic interactions with the metal cations [22]. This possible mechanism helps explain the improved adsorption performance observed at higher pH values.

The effect of temperature on the adsorption of Pb^2+^, Cu^2+^, Cd^2+^, and Ni^2+^ by the FAGP composite fibers is presented in Figure 6d. When the temperature increased from 25 °C to 45 °C, the uptake efficiencies of Pb^2+^, Cu^2+^, Cd^2+^, and Ni^2+^ increased to 100%, 69.14%, 80.34%, and 42.79%, respectively. The observed increase in adsorption capacity at higher temperatures may be partially attributed to enhanced ion mobility and diffusion rates, which improve access to adsorption sites. A secondary hypothesis is that thermal effects can induce microstructural rearrangements such as the formation or widening of microcavities within the PES–geopolymer matrix, thereby exposing additional active sites. This notion is supported by studies showing that varying curing or exposure temperatures can alter porosity and pore connectivity in geopolymers [35].

The efficient heavy-metal adsorption on the FAGP composite fibers under optimal adsorption conditions indicates their high potential for industrial wastewater treatment applications. The efficient removal of Pb^2+^, Cu^2+^, Cd^2+^, and Ni^2+^ at pH = 5 suggests that these fibers are suitable for wastewater streams with moderate acidity, minimizing the need for extensive pH adjustment. The temperature-responsive adsorption behavior further highlights the suitability of these materials for treating high-temperature industrial effluents, utilizing waste heat to enhance performance. Although the removal efficiency decreases at high metal-ion concentrations, the composite fibers remain effective for treating wastewaters containing low metal-ion concentrations, which is typical of several industrial discharges. Overall, these findings establish a promising basis for integrating FAGP composite fibers into large-scale, sustainable wastewater treatment systems.

### 3.3. Isotherm Model Analysis

The adsorption isotherms of Pb^2+^, Cu^2+^, Cd^2+^, and Ni^2+^ on FAGP composite fibers were assessed using five adsorption isotherm models: Langmuir, Freundlich, Dubinin–Radushkevich (D–R), Redlich–Peterson (R–P), and Temkin [36,37,38,39]. These five models were applied to fit the experimental data obtained in this work. The maximal capacities of the FAGP composite fibers for Pb^2+^, Cu^2+^, Cd^2+^, and Ni^2+^ were determined based on the experimental results. The adsorption isotherms for the removal of Pb^2+^, Cu^2+^, Cd^2+^, and Ni^2+^ were studied at different initial metal-ion concentration of the solutions, an adsorbent amount of 0.1 g, a temperature of 25 °C, and a solution pH of 5. The obtained data were then fitted to the Langmuir, Freundlich, D–R, R–P, and Temkin models.

The Langmuir isotherm is one of the most common isotherm models that are valid for monolayer adsorption on an adsorbent surface. The linear form of this equation is expressed by Equation (4).

(4) $$\frac{Ce}{qe}=\frac{1}{qmK_{L}}+\frac{Ce}{q_{m}},$$

(5) $$R_{L}=\frac{1}{1+K_{L}C_{o}},$$

where *Ce* is the equilibrium concentration of heavy-metal ions (mg/L), *qe* is the amount of heavy-metal ions adsorbed per unit mass of the adsorbent (mg/g), *K_L_* is the Langmuir adsorption constant (L/mg), *q_m_* is the maximum amount per unit mass of adsorbent to form a complete monolayer on the surface (mg/g), *C_O_* is the initial concentration of heavy-metal ions (mg/L) and *R_L_* is separation factor. The value of Rᴸ indicates the type of isotherm and the nature of the adsorption process: *R_L_* > 1 represents unfavorable adsorption, *R_L_* = 1 denotes linear adsorption, 0 < *R_L_* < 1 indicates favorable adsorption, and *R_L_* = 0 suggests irreversible adsorption.

The Freundlich isotherm is used for multilayer adsorption and is related to heterogeneous surfaces, and it is expressed by Equation (6).

(6) $$ln(q_{e})=ln(K_{F})+\frac{1}{n}lnCe,$$

where *q_e_* is the quantity of the solute adsorbed per unit mass of the adsorbent (mg/g), *C_e_* is the equilibrium concentration of the adsorbent (mg/L), *K_F_* is the adsorption capacity when the metal-ion equilibrium concentration is equal to 1, and *n* is the degree of dependence of adsorption on the equilibrium concentration.

The R–P adsorption isotherm model is an empirical isotherm; therefore, the adsorption mechanism does not follow an ideal monolayer adsorption. Moreover, this adsorption isotherm is a combination of Langmuir isotherm and Freundlich isotherm. The linear form of this formulation is expressed by Equation (7).

(7) $$ln\frac{Ce}{q_{e}}=glnc_{e}-ln\;K_{RP},$$

where *qe* is the quantity of the solute adsorbed per unit mass of the adsorbent (mg/g), *Ce* is the equilibrium concentration of the adsorbent (mg/L), *K_RP_* is the Redlich constant (L/g) and *g* is an exponent (*g* = 0–1).

The D–R adsorption isotherm is also an empirical adsorption model that does not assume a homogeneous surface or constant adsorption potential. This model is more general than the Langmuir isotherm and is used to describe physical adsorption. The D–R isotherm provides insight into the mean adsorption energy (E), where values of E < 8 kJ/mol indicate physisorption, values between 8 and 16 kJ/mol suggest ion-exchange mechanisms, and values > 16 kJ/mol are typically associated with chemisorption. The D–R model is expressed by Equations (8)–(10).

ln*q_e_* = ln*q_m_* − βε^2^ (8)

(9) $$\varepsilon =RTln\left(1+\frac{1}{C_{e}}\right),$$

(10) $$E=\frac{1}{{2\beta }^{0.5}},$$

where q_e_ is the quantity of the solute adsorbed per unit mass of adsorbent (mg/g), q_m_ is the adsorption capacity (mol/g), β is the D–R constant (mol^2^/kJ), ε is the Polanyi potential, R is the ideal gas constant (8.314 J/mol-k), T is the absolute temperature (K), and E is the mean free energy of adsorption (kJ/mol).

Finally, the Temkin adsorption isotherm accounts for interactions between adsorbed species. Unlike models that assume a logarithmic decline, the Temkin model proposes that the heat of adsorption decreases linearly with increasing surface coverage at intermediate concentrations. It further assumes a uniform distribution of binding energies up to a maximum value. The model is expressed as:

(11) $$q_{e}=\frac{R_{T}}{b}ln(A_{T}C_{e}),$$

(12) $$q_{e}=\frac{R_{T}}{b_{T}}ln(A_{T})+\left[\frac{R_{T}}{b}\right]lnC_{e},$$

(13) $$B=\frac{R_{T}}{b_{T}},$$

q_e_ = B ln A_T_ + B ln C_e_, (14)

where A_T_ is the Temkin equilibrium binding constant (L/g), B_T_ is the Temkin constant, R is the universal gas constant (8.314 J/mol-k), T is the absolute temperature (298 K), and B represents the heat of adsorption (J/mol).

**Figure 7:**
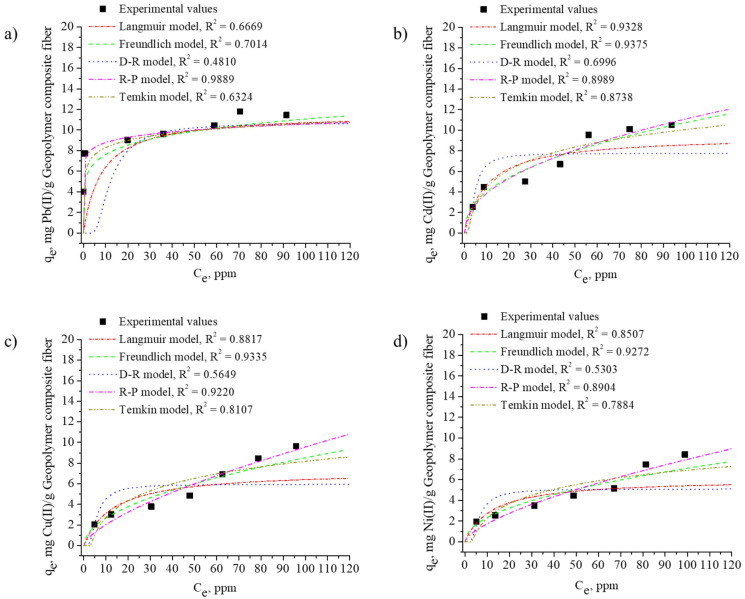
Adsorption isotherms of (**a**) Pb^2+^, (**b**) Cd^2+^, (**c**) Cu^2+^, and (**d**) Ni^2+^ obtained by applying the Langmuir, Freundlich, Redlich–Peterson, Dubinin–Radushkevich, and Temkin isotherm models to the FAGP composite fibers.

Table 3 presents the calculated parameters and correlation coefficients for the five adsorption isotherm models, while the corresponding plots are shown in Figure 7. As illustrated in Figure 7a, the R–P adsorption isotherm, which integrates the features of both the Langmuir and Freundlich models, exhibited the highest correlation (R^2^ = 0.9889) for Pb^2+^ adsorption on the FAGP composite fibers. This result suggests that Pb^2+^ can be adsorbed on fiber surfaces through a combination of monolayer and multilayer mechanisms.

In addition, the Freundlich adsorption isotherm demonstrated strong correlations for the Cd^2+^, Cu^2+^, and Ni^2+^ adsorption, with R^2^ values of 0.9375, 0.9335, and 0.9272, respectively (Figure 7b–d). These findings indicate that Cd^2+^, Cu^2+^, and Ni^2+^ were primarily adsorbed in multilayer arrangements on the heterogeneous surfaces of the FAGP composite fibers. The maximum adsorption capacities for Pb^2+^, Cd^2+^, Cu^2+^, and Ni^2+^ were 11.78, 10.79, 9.64, and 8.42 mg/g, respectively.

The adsorption equilibrium data were analyzed using the Langmuir, Freundlich, Temkin, Redlich–Peterson, and Dubinin–Radushkevich (D–R) isotherm models to better understand the adsorption mechanisms of Pb^2+^, Cd^2+^, Cu^2+^, and Ni^2+^ on the FAGP composite fibers. Among the tested models, the adsorption of Pb^2+^ was best described by the Redlich–Peterson isotherm, indicating a combination of monolayer and heterogeneous adsorption behavior. In contrast, the adsorption of Cd^2+^, Cu^2+^, and Ni^2+^ fitted better with the Freundlich isotherm, suggesting multilayer adsorption on heterogeneous surfaces.

The Langmuir separation factor (R_L_), derived from the Langmuir constant (K_L_) and the initial concentration (C_O_), and was used to evaluate the favorability of the adsorption process. The calculated Rᴸ values for Pb^2+^, Cd^2+^, Cu^2+^, and Ni^2+^ were 0.082, 0.098, 0.120, and 0.110, respectively, all within the range of 0 < R_L_ < 1, confirming favorable adsorption behavior. These favorable Rᴸ values are consistent with the Temkin and D–R isotherm results, which further highlight strong adsorbate–adsorbent interactions. The Temkin model yielded relatively high B constants, while the D–R model produced mean adsorption energy (E) values exceeding 16 kJ/mol, indicating that chemisorption predominates. Overall, the adsorption of heavy-metal ions onto the FAGP composite fibers proceeds mainly through chemisorption with contributions from ion exchange and surface complexation.

### 3.4. Kinetics and Intraparticle Diffusion

The adsorption mechanism can be further elucidated through the application of kinetic models. Herein, kinetic analyses were conducted to investigate the roles of external and internal diffusion as well as the chemical reaction mechanisms during the adsorption process. To gain insights into the underlying mechanisms of the adsorption process, time-dependent experimental data were fitted using the Weber–Morris intraparticle diffusion model as well as the pseudo-first-order and pseudo-second-order kinetic models [9,14,40]. The Weber–Morris intraparticle diffusion model was specifically employed to compare the saturation times of different metal ions on the composite fibers. The mathematical expression of the Weber–Morris intraparticle diffusion model is given by Equation (15).

*qt = K_p_t*^1/2^* + C*, (15)

where *qt* is the adsorption capacity at any time (mg/g), *K_p_* is the intraparticle diffusion rate constant (mg/g h^1/2^), *t* is time (h), and *C* is the intercept of the linear graph (mg/g).

**Figure 8:**
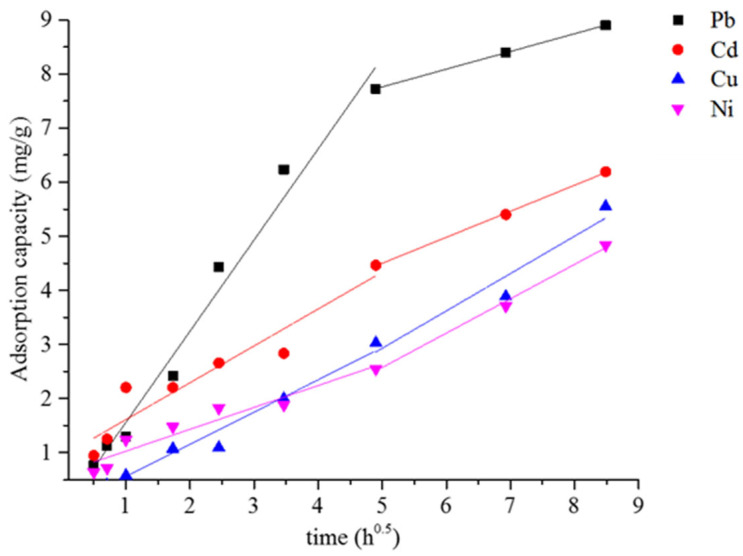
The Weber–Morris intraparticle diffusion plots of the heavy-metal ions on the FAGP composite fibers.

Typically, an intraparticle diffusion plot can be divided into three distinct regions. These regions correspond to the diffusion of ions to the external surface of the adsorbent; the intraparticle diffusion, where ions migrate into the internal pores of the adsorbent; and the equilibrium phase, during which the adsorption sites become saturated with ions [41]. The intraparticle diffusion profiles of each metal-ion on the FAGP composite fibers are shown in Figure 8. Only two distinct regions were observed in the intraparticle diffusion plots of all examined metal ions on the FAGP composite fibers. The first region corresponds to the adsorption and diffusion of metal ions on the surface of the composite fibers and into the PES matrix. The second region reflects the diffusion of metal ions into the pores of the FAGP powder, which is the dominant mechanism governing the overall adsorption process. The intraparticle diffusion parameters for Pb^2+^, Cd^2+^, Cu^2+^, and Ni^2+^ adsorption are summarized in Table 4. Among the examined ions, Pb^2+^ exhibited the highest intraparticle diffusion rate constant (K_p_) in the first region, indicating that it has the fastest diffusion into the composite fibers. The order of diffusion rates was as follows: Pb^2+^ > Cd^2+^ > Cu^2+^ > Ni^2+^, which is in good agreement with the order of the adsorption capacities.

In addition, the kinetics of metal-ion adsorption on the FAGP composite fibers were analyzed using the pseudo-first-order model, pseudo-second-order model and Elovich kinetic model. The kinetic parameters of the adsorption process were measured based on a batch adsorption experiment at 25 °C and pH = 5, and the results were fitted to the pseudo-first-order rate equation (Equation (16)).

*ln(q_e_ − q_t_) = lnq_e_ − k*_1_*t*, (16)

In the pseudo-second-order model, if the linearized rate of adsorption follows a second-order chemisorption kinetics, the equation is expressed by Equation (17).

(17) $$\frac{t}{q_{t}}=\frac{1}{k_{2}q_{e}^{2}}+\frac{1}{q_{e}}t,$$

where *qe* is the adsorption capacity at equilibrium (mg/g), *qt* is the adsorption capacity at any time (mg/g), *t* is time (min), *k*_1_ is the rate constant of pseudo-first-order sorption (min^−1^), and *k*_2_ is the rate constant of pseudo-second-order sorption (g/mg min).

Moreover, the Elovich kinetic model is commonly applied to describe adsorption processes occurring on heterogeneous surfaces, particularly when chemisorption is involved. The model assumes that the rate of adsorption decreases exponentially with increasing surface coverage, reflecting a multilayer or energetically non-uniform surface. The linear form of the Elovich equation can be expressed as (Equation (18)):

(18) $$q_{t}=\frac{1}{\beta }ln(\alpha \beta )+\frac{1}{\beta }ln(t),$$

where q_t_ (mg/g) is the amount of adsorbate adsorbed at time *t*(min), *α* (mg/g·min) is the initial adsorption rate, and *β*(g/mg) is the desorption constant related to surface coverage and activation energy for chemisorption.

**Figure 9:**
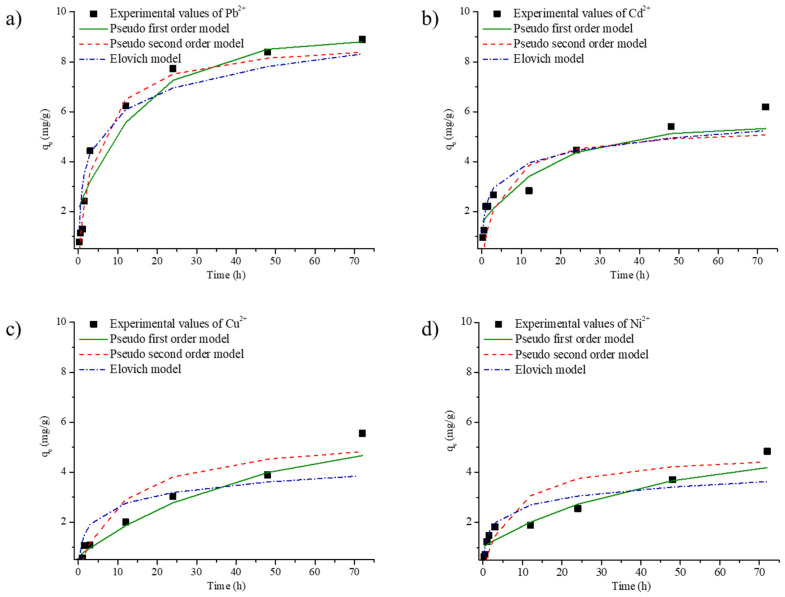
Kinetics of (**a**) Pb^2+^, (**b**) Cd^2+^, (**c**) Cu^2+^, and (**d**) Ni^2+^ adsorption on the FAGP composite fibers and the fit of the model to the data for mono-cation solutions (pseudo-first-order model, pseudo-second-order model, and Elovich model).

The experimental adsorption data for Pb^2+^, Cd^2+^, Cu^2+^, and Ni^2+^ on the FAGP composite fibers were analyzed using pseudo-first-order, pseudo-second-order and elovich kinetic models (Figure 9). Moreover, the experimental adsorption capacities (qₑ,exp) together with the model-calculated values for each kinetic equation, enabling direct comparison between experimental and predicted results are summarized in Table 5. Based on the linear regression coefficients (R^2^ values), the pseudo-second-order model provided a better description of the adsorption mechanism for Pb^2+^, whereas the pseudo-first-order model more accurately described the adsorption behaviors of Cd^2+^, Cu^2+^, and Ni^2+^. Nevertheless, the experimentally determined adsorption capacities were generally closer to the values predicted by the pseudo-second-order model, with discrepancies of 10–20%. Overall, the kinetic analysis suggests that the pseudo-second-order model offers a more reliable prediction of the adsorption kinetics for all studied metal ions, particularly for Pb^2+^ adsorption on the FAGP composite fibers.

The adsorption isotherms and kinetics indicate that the adsorption process involves both monolayer and multilayer interactions depending on the metal ion. The R–P model revealed that the Pb^2+^ adsorption involves both monolayer and multilayer adsorption. In contrast, the Freundlich model indicated that the Cu^2+^, Cd^2+^, and Ni^2+^ adsorption processes proceed through a multilayer adsorption on a heterogeneous surface. The pseudo-second-order kinetics of Pb^2+^ reflected a chemisorption or ion exchange mechanism, in which the adsorption depends on the chemical bonds between the adsorbent surface and the metal ions, indicating strong interactions. In contrast, the pseudo-first-order kinetics of Cu^2+^, Cd^2+^, and Ni^2+^ indicated that their adsorption proceed through a combination of surface adsorption and diffusion, which may involve weaker physical interactions (physisorption). This indicates that the adsorption of different metal ions on the composite fibers occurs through a combination of both chemisorption and physisorption, with interactions of varying strengths depending on the ion.

Table 6 compares the adsorption capacities of the FAGP composite fibers (60 wt% FAGP) for Pb^2+^, Cd^2+^, Cu^2+^, and Ni^2+^ with those of other reported adsorbents, including magnetic activated carbon powders, natural clays, metakaolin-based geopolymer powders, geopolymer foams, and geopolymer spheres. The FAGP fibers exhibited adsorption capacities of 11.78, 10.79, 9.64, and 8.42 mg/g for Pb^2+^, Cd^2+^, Cu^2+^, and Ni^2+^, respectively. Although these values are lower than those of magnetic activated carbon (253.20 mg/g for Pb^2+^) and metakaolin-based geopolymers (312.50 mg/g for Pb^2+^), they are comparable to or even exceed those of natural clays and geopolymer foams for certain ions, particularly Cd^2+^ and Cu^2+^. For example, the FAGP fibers achieved higher Cd^2+^ removal than geopolymer foams (2.81 mg/g for Cd^2+^) and better overall performance for multiple ions than Ni^2+^-selective geopolymer spheres (19.94 mg/g for Ni^2+^).

While magnetic activated carbons and metakaolin-based geopolymers demonstrate higher maximum adsorption capacities, they often involve higher production costs or raw material limitations. In contrast, the FAGP composite fibers provide a cost-effective, sustainable, and environmentally friendly alternative, as they valorize industrial fly ash while maintaining balanced adsorption efficiency across multiple heavy metals. Importantly, the novelty of this study lies not in capacity alone but in the fiber format, which offers handling, separation, and scalability advantages that powders, foams, or spheres cannot easily deliver. Therefore, the key contribution of this work is the demonstration of a scalable, mechanically stable, and environmentally sustainable fiber-based geopolymer adsorbent that bridges laboratory-scale studies with potential practical applications in wastewater treatment.

## 4. Conclusions

This study developed fly ash-based geopolymer (FAGP) composite fibers reinforced with polyethersulfone (PES) for the adsorption of Pb^2+^, Cd^2+^, Cu^2+^, and Ni^2+^ ions from aqueous solutions. The incorporation of FAGP within the PES matrix produced fibers with uniform particle dispersion and improved structural integrity, as confirmed by SEM and X-ray CT analysis.

Adsorption performance was strongly influenced by operational parameters such as pH, contact time, and adsorbent dosage. The optimal condition achieved complete Pb^2+^ removal (100%) at 0.5 g dosage and pH 5, with significant removal of Cd^2+^, Cu^2+^, and Ni^2+^ under the same conditions. Isotherm analysis revealed that Pb^2+^ adsorption followed the Redlich–Peterson model, indicating a combination of monolayer and heterogeneous adsorption, while Cd^2+^, Cu^2+^, and Ni^2+^ were better fitted by the Freundlich model, suggesting multilayer adsorption. The favorable Langmuir separation factors (0 < R_L_ < 1) confirmed that adsorption was spontaneous and efficient. The Temkin and Dubinin–Radushkevich (D–R) models further supported strong adsorbate–adsorbent interactions, with D–R mean adsorption energy (E) values above 16 kJ/mol, confirming chemisorption as the dominant mechanism.

Overall, the FAGP composite fibers demonstrated high adsorption efficiency, good mechanical stability, and scalability potential, making them promising materials for wastewater treatment applications. The use of fly ash as a raw material enhances sustainability and cost-effectiveness. Future research will focus on multicomponent and real wastewater systems, regeneration and reuse cycles, and advanced surface modifications to improve selectivity and long-term performance.

## Figures and Tables

**Table 1:** Chemical compositions of the composite fibers, PES, and FAGP powders.

Chemical Compound (%)	PES	Washed FAGP Powder	20 wt% FAGP Fiber	40 wt% FAGP Fiber	60 wt% FAGP Fiber
SiO_2_	-	44.80	5.44	25.30	42.40
Al_2_O_3_	-	16.30	2.13	6.36	9.89
Fe_2_O_3_	-	12.40	1.15	3.89	6.39
CaO	-	7.64	0.44	1.65	2.65
MgO	-	1.49	0.16	0.57	0.77
SO_3_	20.70	0.10	20.30	18.10	13.20
Na_2_O	-	0.80	0.12	0.23	0.42
CO_2_	79.10	13.80	69.90	52.40	40.00

**Table 2:** Properties of the composite fibers, PES, and FAGP powders.

Samples	Obvious Specific Volume (cm^3^/g)	Tensile Strength (MPa)	Surface Area (m^2^/g)
PES	0.70	5.83	27.39
20 wt% FAGP	0.62	4.17	50.05
40 wt% FAGP	0.60	2.35	57.50
60 wt% FAGP	0.53	1.40	71.67
FAGP powder	-	-	85.01

**Table 3:** Parameters of the Langmuir, Freundlich, Redlich–Peterson, Dubinin–Radushkevich, and Temkin isotherms.

Isotherm Model	Parameter	Metal			
		Pb^2+^	Cd^2+^	Cu^2+^	Ni^2+^
Langmuir	q_m_	11.574	9.343	7.246	6.068
	K_L_	0.123	0.098	0.076	0.082
	R^2^	0.6669	0.9328	0.8817	0.8507
	R_L_	0.082	0.098	0.120	0.110
Freundlich	K_F_	5.249	1.497	0.835	0.762
	1/n	0.162	0.428	0.505	0.485
	R^2^	0.7014	0.6996	0.9335	0.9272
Redlich–Peterson (R–P)	K_RP_	8.40 × 10^6^	27,493.8	11,319.9	5640.06
	a	1.15 × 10^6^	20,586.1	26,018	14,686.6
	g	0.920	0.540	0.329	0.342
	R^2^	0.8289	0.8989	0.9220	0.8904
Dubinin–Radushkevich (D–R)	q_m_	10.710	7.752	5.964	5.105
	β	2 × 10^−5^	3 × 10^−6^	5 × 10^−6^	6 × 10^−6^
	R^2^	0.4810	0.6996	0.5649	0.5303
	E	158.114	408.248	316.228	288.675
Temkin	A_T_	1.002	0.998	0.997	0.998
	b	2433.316	1010.196	1042.002	1230.977
	B	1.018	2.453	2.378	2.013
	R^2^	0.6324	0.8738	0.8107	0.7884
q_e_ of Experimentally value (mg/g)	q_e_	11.78	10.79	9.64	8.42

**Table 4:** The Weber–Morris intraparticle diffusion parameters for metal-ion adsorption on the FAGP composite fibers.

Metal	Intraparticle Diffusion		
	k_p_ (mg/g h^0.5^)	C (mg/g)	R^2^
Pb^2+^	1.684	0.1204	0.9907
Cd^2+^	0.681	0.932	0.9527
Cu^2+^	0.596	0.034	0.9869
Ni^2+^	0.404	0.063	0.9618

**Table 5:** Parameter values for the batch kinetic adsorption models of the FAGP composite fibers.

Kinetic Model	Parameter	Metal			
		Pb^2+^	Cd^2+^	Cu^2+^	Ni^2+^
Pseudo-first order	k_1_ (min^−1^)	0.0001	0.0001	0.0003	0.0004
	q_m_ (mg/g)	6.794	3.856	4.908	3.806
	R^2^	0.9501	0.8960	0.9775	0.9506
Pseudo-second order	k_2_ (g/mg min)	0.0004	0.0005	0.0002	0.0005
	q_m_ (mg/g)	9.327	6.208	5.550	4.670
	R^2^	0.9977	0.6878	0.9125	0.9157
Elovich	β	0.8030	1.3869	1.6339	1.9409
	α	0.2295	0.2395	0.0772	0.1347
	R^2^	0.8966	0.8759	0.7779	0.8380
q_e_ of Experimentally value (mg/g)	q_e_	8.899	6.192	5.551	4.840

**Table 6:** Comparison between the adsorption capacities of the FAGP composite fibers and other reported heavy-metal-ion adsorbents.

Adsorbent Materials	Metal Ion	Adsorption Capacity (mg/g)	References
FAGP Composite Fibers	Pb^2+^	11.78	This study
(60 wt%)	Cd^2+^	10.79	
	Cu^2+^	9.64	
	Ni^2+^	8.42	
Magnetic activated carbon	Pb^2+^	253.20	[42]
powder	Cd^2+^	73.30	
Natural clay powder	Pb^2+^	9.91	[43]
	Cd^2+^	9.45	
	Ni^2+^	10.20	
Metakaolin-based	Pb^2+^	312.50	[44]
Geopolymers powder	Cu^2+^	178.60	
Geopolymer foams	Pb^2+^	11.99	[45]
	Cd^2+^	2.81	
	Ni^2+^	6.16	
Geopolymer spheres	Ni^2+^	19.94	[46]

## Data Availability

The original contributions presented in this study are included in the article. Further inquiries can be directed to the corresponding author.
